# Letter to editor: Complementary perspectives on B cell regulatory phenotypes in cancer

**DOI:** 10.3389/fimmu.2025.1647502

**Published:** 2025-08-19

**Authors:** Akshay J. Patel, Gary W. Middleton

**Affiliations:** Institute of Immunology and Immunotherapy (III), College of Medical and Dental Sciences, University of Birmingham, Birmingham, United Kingdom

**Keywords:** Breg, lung cancer, non-small cel lung carcinoma, vista, immunotherapy

## Introduction

B cells are increasingly recognised as key regulators of immune responses in cancer, yet their suppressive roles and defining features remain poorly understood. Regulatory B cells (Bregs), which secrete cytokines such as IL-10 and TGF-β, may modulate the tumour microenvironment and influence outcomes of immunotherapy. In a recent study, Lo Tartaro et al. identified a novel subset of intratumoral Bregs expressing the immune checkpoint molecule VISTA, with distinct transcriptional and metabolic features in early-stage NSCLC. However, functional comparisons to previously reported Breg phenotypes were not explored.

Our earlier work has characterised Breg developmental pathways and cytokine polyfunctionality in lung cancer patients, with clear links to immunotherapy-related toxicity. In this commentary, we provide complementary insights to support and extend the findings reported by Lo Tartaro et al.

## Commentary

In recent years, the immunoregulatory functions of B cells within the TME have garnered increasing attention, particularly in the context of their contribution to immune evasion and therapeutic resistance. Bregs, a phenotypically and functionally heterogeneous subset, have emerged as pivotal mediators of immune suppression, primarily through IL-10 (their primary hallmark), TGF-β and IL-35 secretion (1–3) and modulation of T cell responses. However, the specific molecular features that define immunosuppressive Bregs in human cancers remain poorly understood.

We read with great interest the recent publication by Lo Tartaro et al. investigating the role of VISTA+ regulatory B cells (Bregs) within the tumour microenvironment (TME) (4). Their study provides a comprehensive transcriptomic and spatial characterisation of this B cell subset in non-small cell lung cancer (NSCLC), revealing a population of CD27+CD38^hi^CD24- plasmablast-like cells enriched for the immune checkpoint molecule VISTA. Through integrative single-cell RNA sequencing and spatial transcriptomic approaches, the authors identify a VISTA+ B cell population with an immunoregulatory gene signature and enrichment for suppressive cytokines and checkpoint markers, suggesting a novel immunosuppressive role within the tumour niche.

The diffusion map trajectory analysis (Figure 1G) suggests that VISTA^+^ B cells occupy a transcriptionally distinct continuum suggestive of a regulatory trajectory, diverging from naïve and memory B cell subsets. These cells are enriched for gene signatures associated with immune regulation and suppression and are proposed to exert their function via soluble factor secretion rather than antigen-specific interactions. Our earlier work (1) has demonstrated the same distinct developmental trajectories that Bregs can take (Figure 2B), showing clear separation between the plasmablast phenotype (Ki67^hi^ CD38^hi^ CD27^hi^ CD95^hi^ IL-10^int^) and the PD1^hi^ and PD-L1^hi^ TGFβ+ populations. The clear lack of phenotypic surrogacy is nicely shown by this group (4) between blood and tumour tissue compartments, evident by the contrasting diffusion maps between the two regions. Further work from our group (5) has again corroborated these findings; Figure 1E shows a notable difference in B and Plasma cell trajectory between tumour and blood. The significantly higher presence of CD19^lo^ CD38^hi^ CD24- CD27^lo^ IgD- antibody secreting plasma cells in the tumour compared to blood, and the contrasting abundance of naïve, memory and plasmablast cells in circulation was a key finding from our work.

Figure 3F (4) very nicely delineates the clear polyfunctional cytokine expression between VISTA+ and VISTA- Bregs. The positive cells, express a range of pro-inflammatory and anti-inflammatory cytokines which is lost in VISTA- populations. Drawing parallels with our work (1), from Figure 1C, we have demonstrated a range of cytokine induction (IL-10, IFNγ, TNFα, IL-17, IL-6 and IL-2) between healthy controls and Bregs taken from lung cancer patients who do and do not develop autoinflammatory sequelae post treatment with checkpoint blockade. Healthy controls and patients who do not develop post-treatment toxicity tend to display a normal range of cytokine production whereas patients who develop post-treatment toxicity have a polyfunctional Breg failure suggesting a germline or perhaps an early developmental block in the ability to mount a cytokine response to the appropriate environmental stimuli. Based on this insightful work, VISTA presence may be a pivotal factor in determining the functional maturity of Bregs *in vivo*, and furthermore whether it could act as a biomarker for Bregs, akin to Foxp3 on Tregs, which hitherto has not been shown in Breg biology.

Despite these insights, the study does not experimentally validate the functional capacity of VISTA^+^ Bregs, nor does it directly explore their impact on T cell effector function or clinical outcomes following checkpoint blockade. Hence, whether VISTA blockade abrogates polyfunctional cytokine expression ex vivo remains to be seen. Nevertheless, the work adds to the growing body of literature identifying immunosuppressive B cell subsets in human cancers and proposes VISTA as a novel surface marker that may aid in the delineation of regulatory B cell states.
